# Artificial intelligence investigation of magneto radiated nanofluid under mixed convection

**DOI:** 10.1186/s11671-026-04688-2

**Published:** 2026-06-05

**Authors:** Adnan, Ghulfam Sarfraz, Mohamed Abubakar Fiidow, Mohamed Arbi Khlifi, Mustafa Abdullah, Refka Ghodhbani, Samia Elattar, Muhammad Nasir Bashir

**Affiliations:** 1 Department of Mathematics, Mohi-ud-Din Islamic University, Nerian Sharif, AJ&K, 12080 Pakistan; 2 Department of Mathematical Sciences, Faculty of Science, Somali National University, Mogadishu Campus, Mogadishu, Somalia; 3 Department of Electrical Engineering, Faculty of Engineering, Islamic University of Madinah, Madinah, 42351 Saudi Arabia; 4 Electric Vehicles Engineering Department, Faculty of Engineering, Hourani Center for Applied Scientific Research, Al-Ahliyya Amman University, Amman, Jordan; 5 Center for Scientific Research and Entrepreneurship, Northern Border University, Arar, 73213 Saudi Arabia; 6 Department of Industrial and Systems Engineering, College of Engineering, Princess Nourah bint Abdulrahman University, P.O. Box 84428, Riyadh, 11671 Saudi Arabia; 7 Department of Mechanical Engineering, College of Engineering, King Faisal University, Al-Ahsa, 31982 Saudi Arabia; 8 Department of Mechanical Engineering, University of Malaya, 50603 Kuala Lumpur, Malaysia

**Keywords:** Solar radiations, Nanofluids, AI approach, Dissipation effects, Mixed convection

## Abstract

Thermal radiations study for heat transfer applications using AI based algorithm known as Levenberg Marquardt Back Propagation Method (LMBPM) is an effective area. Hence, the current problem describes the effective performance of nanofluid through the LMBPM. The flow scenario is considered for plate which placed horizontally in the cartesian system. The $\:A{l}_{2}{O}_{3}$ and water are the functional fluid components in the absence of chemical reactions. The model obtained using the thermophysical relations and transformative functions in the presence of dissipation effects, solar radiations and combined convection. Further, the results furnished using 10 neurons in hidden while 5 neurons in output layer and computed the physical outcomes. The study’s findings reveal that the gravity effect depreciated the thermal boundary layer while the Lorentz forces due to normally acting magnetic field opposes the nanofluid motion. Thermal radiations and Eckert number provided considerable improvement in the temperature. The accuracy and validity of the scheme is studied through validations checks, fitting functions, histogram and regression analysis and found excellent outcomes. The conducted study along with influential physical controls will help to manage the heat transfer with suggested parametric ranges. These will practically applicable in engineering systems particularly in heating and cooling, thermal transport in nano-devices and heat exchangers applications.

## Introduction

Nanofluids playing crucial role in the heat transfer applications. These consist of variety of nanoparticles and base liquids. In recent years, the trends have been observed towards the analysis of nanofluids from multiple aspects using stretchable surfaces. Ali et al. [1] examined an unsteady nanoliquid problem through a sheet and summarized that the rough surface has influential effects on the velocity. Also, the constant flow pattern has been examined in response of injecting fluid. Alam et al. [2] studied the porosity influences on the flow problem with changeable thickness of the surface. They reported that both thermal and mass performance improved as the thermophoresis force enlarges. More accumulation of nanomaterials in base fluid enhanced the temperature distribution. Irfan et al. [3] considered the flow over a surface with varying thickness and revealed that the constant fluid properties have less effects on the fluid dynamics. Liu et al. [4] evaluated the heat transfer in the presence of stretching surface effects and evidenced that TBL are thicker for higher fractional parameter.

An EMHD flow of nanofluid along with slandering sheet was reconnoitered by Ali et al. [5]. They noted that the higher impacts of magnetic forces are responsible to declines the movement. On the other hand, the temperature distribution improved due to increasing the electric field. Pattnaik et al. [6], and Dey et al. [7] conducted studies for deep thermal mechanism in Carreau type ternary and single phase hybrid nanoliquid suspended by different NPs structures. The analysis shows significant impacts of the parameters and their momentum and thermal controlling capacity and highlighted their applications in industries. Panda et al. [8] explored that ferrite nanoparticles have remarkable ability to improve the heat mechanism due to modified properties of base liquid. The results presented through bvp4c algorithm and also discussed the tabulated data for engineering quantities. AI schemes observed to be very influential in recent time to estimate the heat transfer in variety of fluids and compared with other schemes. In this regard, a significant analysis has been provided in [9]. Multiple aspects of ternary nanoliquids using two distinct configurations with varying parameters have been reported in the Refs. [10] and [11], respectively. Both investigations useful insights in the use of hybrid nanoparticles for improved dynamics of primary solvents.

The hydromagnetic nanofluid under heat flux and dissipative energy effects were discussed by Ghoneim et al. [12]. They investigated that the cooling of sheet improved due to higher Brownian motion. Prasad et al. [13] examined magnetized nanoliquid properties and noticed that the flow decreased in suction case whereas increased in injection. Sreelakshmi et al. [14] discussed characteristics of inertial forces on micropolar fluid in 3D. The study provided deep impacts of time dependent heat source by accommodating the convection effects and found strong bonding of these situations for heat transfer. Awais et al. [15] explored the importance of Fourier flux with combination of Au and Ag nanomaterials that consistently dispersed in Sutterby fluid. The model discussed through RK 4th order algorithm [16] and examined high contribution of these particles in the problem results at multiple concentrations. Time dependent flows through stagnant point are crucial from engineering point of view. A useful study revealing the impacts of nonlinear radiative heat flux on engine oil under unsteady situation is reported in [17]. Combination of various nanomaterials is a good technique to boost the efficiency of traditional fluids. Additional physical impacts within or from the surrounding playing critical role on the fluids system. Thus, important studies in this regard have been described in the Refs. [18] and nonlinear stretchy surface problem analyzed in [19].

Aamir et al. [20] scrutinized the hall impacts on MHD flow using the hybrid nanofluid. They declared that the Hall current influences increase the velocity. On the other hand, magnetic effects showed the reverse behavior. The study of hybrid nanofluid inspired by MHD by considering velocity and thermal slip conditions is investigated by Ramzan et al. [21]. They explored that both slip and non-slip conditions are responsible to uplift the hybrid nanofluid velocity. Durgaprasad et al. [22] evaluated the impacts of Brownian movement on 3D nanofluid flow by using the porous surface and concluded increases in thermophoresis effects, increases the TBL.

*Research gap*: The previous studies emphases on the performance of traditional and nanofluids using analytical or classical numerical schemes under certain physical effects. Specifically, collective influence of dissipation energy, thermal radiation and combined convection on the performance of nanofluids for efficient nanomaterials like Al_2_O_3_ remains inadequately discussed. Further, traditional solution techniques are often facing high computational cost with nonlinearities, couple model. Thus, a specific gap exists in the addition of these physical constraints, utilization of data driven method like ANN (Artificial neural networking) base techniques, to precisely the model and improves predictive ability for heat transfer properties in nanofluids system.

*Novelty*: The current research presents an inclusive heat transfer model that simultaneously examines the influence of mixed convection, dissipation energy and thermal radiative flux on water saturated by Al_2_O_3_, providing a more precise and physically improved heat transport model. Further, main novelty of this research lies in the implementation of Artificial NN scheme based on computational cost factor, which make it effective from classical schemes and provides enhanced accuracy and its implementation for nonlinear model. The assortment of Al_2_O_3_ further improves the problem relevance due to their strengthen characteristics, aiding a more efficient assessment of heat transfer. Moreover, hybridization of progressive physical model with machine learning approach presents a noteworthy methodological advancement in the study of heat transfer.

*Contribution*: This research contributes in developing thermofluid problems with integrated effects of convection, viscous dissipation and radiations on the effectiveness of nanofluids. It determines efficiency of NN based schemes in handling tedious heat transfer problems compared to classical schemes. The analysis adds new insights into the role NPs in improving heat transport rate. Further, it provides a dynamic framework that connect fluid problems with intelligence scheme for considerable heat evolution in engineering.

The objectives of this research will to:

- Introduce a nanofluid problem through stretching surface for thermal applications.
- To evaluate the role of combined convection on the velocity of functional nanofluids.
- To analyze the significance of thermal radiations, magnetic field and dissipation energy on the behaviour of nanoliquid.
- To implement the ANN base LMBPS for the problem outcomes by fluctuating the parameters values.

### Model development

**Fig. 1:**
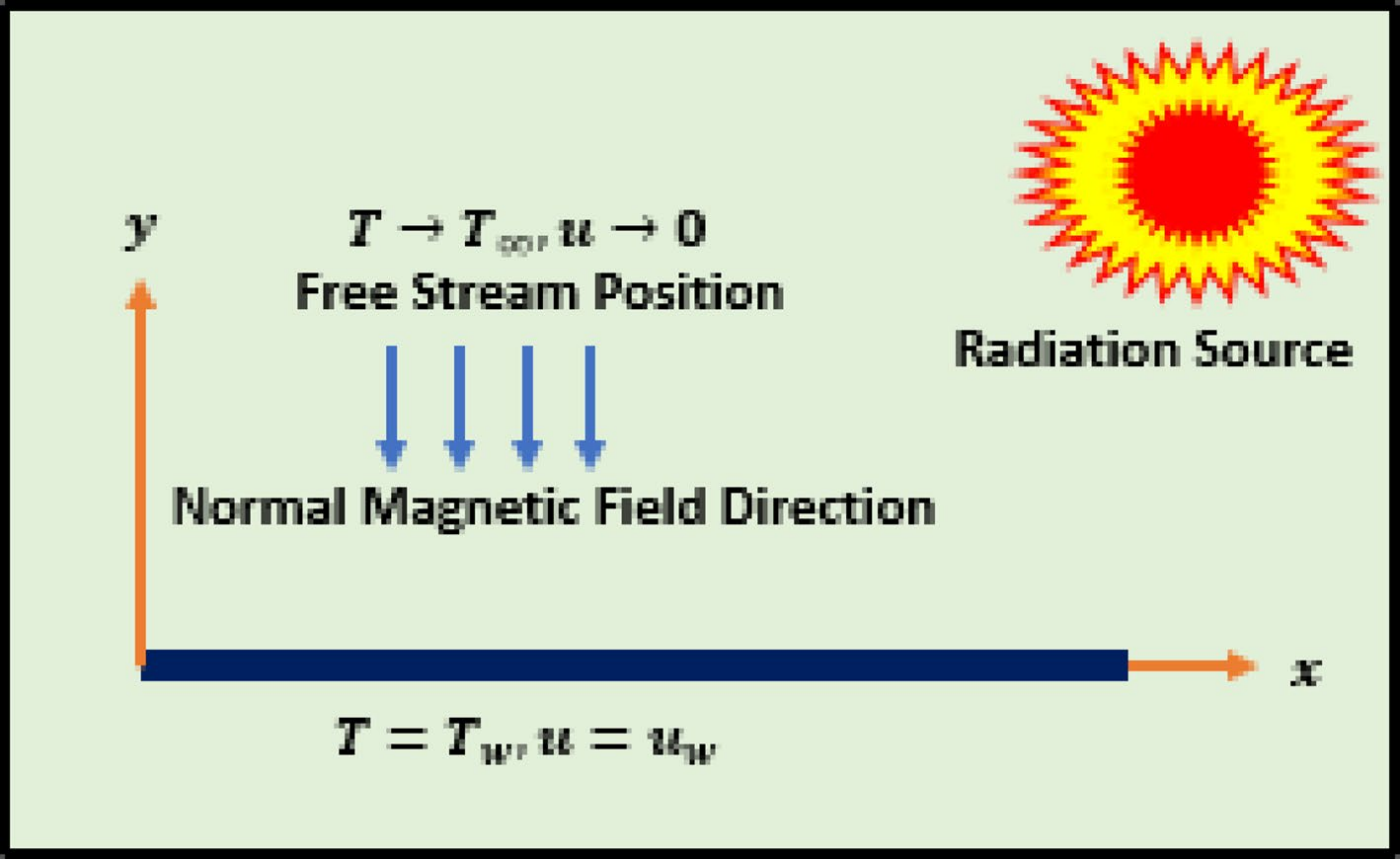
The configuration of nanofluid through a surface

The problem is related to 2D streamlined flow of nanofluid through a horizontally oriented surface. The surface is able to stretch in the direction of $\:x$-axis with $\:u=ax$, the $\:{T}_{w}$ and $\:{T}_{\infty\:}$ designate the fluid temperature at two positions which represents the surface and at free stream, respectively. The fluid flows along $\:x$-axis and the magnetic field acting normally to it. Further, flow accommodates the physical influences of solar radiations, mixed convection, normally imposed magnetic field, and dissipation energy effects. Moreover, the $\:A{l}_{2}{O}_{3}/{H}_{2}O$ fulfills the characteristics of incompressible, homogeneity of the mixture, and viscous. The flow situation is captured in Fig. 1, while rest of the expressions describe the physical laws [23, 24].

1 $$\:\frac{\partial\:u}{\partial\:x}+\frac{\partial\:v}{\partial\:y}=0$$

2 $$\:\left(u\frac{\partial\:u}{\partial\:x}+v\frac{\partial\:u}{\partial\:y}\right){\rho\:}_{nf}={\mu\:}_{nf}\frac{{\partial\:}^{2}u}{\partial\:{y}^{2}}+g{\left(\rho\:\alpha\:\right)}_{nf}\left(T-{T}_{\infty\:}\right)-{\sigma\:}_{nf}{B}_{^\circ\:}^{2}u$$

3 $$\:{(u\frac{\partial\:T}{\partial\:x}+v\frac{\partial\:T}{\partial\:y})\left(\rho\:{c}_{p}\right)}_{nf}=\left({k}_{nf}+\frac{16{\sigma\:}^{*}{T}_{\infty\:}^{3}}{3k{k}^{*}}\right)\frac{{\partial\:}^{2}T}{\partial\:{y}^{2}}+{\mu\:}_{nf}{\left(\frac{\partial\:u}{\partial\:y}\right)}^{2}$$

The conditions are $\:u={U}_{w}=ax,\:v=0,\:T={T}_{w}$ at the surface and $\:u\to\:\infty\:,\:T\to\:{T}_{\infty\:}$ towards the position at free stream. The velocity components $\:u$, $\:v$ and stream-function are expressed the specific flow arrangement as $\:u=\frac{\partial\:\omega\:}{\partial\:y},\:v=-\frac{\partial\:\omega\:}{\partial\:x}$ whereas signifies the stream function and is expressed as.

4 $$\:v=-\sqrt{a{\nu\:}_{f}}F\left(\eta\:\right),\:u=ax{F}^{{\prime\:}}\left(\eta\:\right),\:\eta\:=\sqrt{\frac{a}{{\nu\:}_{f}}}y,\:\omega\:=\sqrt{a{\nu\:}_{f}}xF\left(\eta\:\right),\:\beta\:\left(\eta\:\right)=\frac{T-{T}_{\infty\:}}{{T}_{w}-{T}_{\infty\:}}$$

The properties of traditional fluid augment using the nanomaterial characteristics. The expressions for enhanced thermal model are described as [25].

5 $$\:\left.\begin{array}{c}{\mu\:}_{nf}={\mu\:}_{f}\frac{1}{{\left(1-\phi\:\right)}^{2.5}}\\\:{\rho\:}_{nf}=\left(\left(1-\phi\:\right)+\frac{\phi\:{\rho\:}_{s}}{{\rho\:}_{f}}\right){\rho\:}_{f}\\\:{\left(\rho\:{c}_{p}\right)}_{nf}=\left(\left(1-\phi\:\right)+\frac{\phi\:{\left(\rho\:{c}_{p}\right)}_{s}}{{\left(\rho\:{c}_{p}\right)}_{f}}\right){\left(\rho\:{c}_{p}\right)}_{f}\\\:{\left(\rho\:\alpha\:\right)}_{nf}=\left(\left(1-\phi\:\right)+\frac{\phi\:{\left(\rho\:\alpha\:\right)}_{s}}{{\left(\rho\:\alpha\:\right)}_{f}}\right){\left(\rho\:\alpha\:\right)}_{f}\\\:{k}_{nf}=\left[\frac{\left({k}_{s}+{k}_{f}\right)-2\phi\:\left({k}_{f}-{k}_{s}\right)}{\left({k}_{s}+{k}_{f}\right)+\phi\:\left({k}_{f}-{k}_{s}\right)}\right]\end{array}\right\}$$

Using all of the prescribed information, the resultant problem transforms in the below form.

6 $$\:{F}^{{\prime\:\prime\:\prime\:}}-\frac{{\mu\:}_{f}}{{\mu\:}_{nf}}\left[\frac{{\rho\:}_{nf}}{{\rho\:}_{f}}\left\{{F}^{{{\prime\:}}^{2}}-F{F}^{˶}\right\}-\frac{{\sigma\:}_{nf}}{{\sigma\:}_{f}}\:\frac{{\mu\:}_{f}}{{\mu\:}_{nf}}M{F}^{{\prime\:}}+\frac{{\left(\rho\:\alpha\:\right)}_{nf}}{{\left(\rho\:\alpha\:\right)}_{f}}\:\frac{{\mu\:}_{f}}{{\mu\:}_{nf}}\lambda\:\beta\:\right]=0$$

7 $$\:\left(1+\frac{4}{3}Rd\right){\beta\:}^{˶}+\frac{{\left(\rho\:{C}_{p}\right)}_{nf}}{{\left(\rho\:{C}_{p}\right)}_{f}}\:\frac{{k}_{f}}{{k}_{nf}}\left(PrF\beta\:{\prime\:}\right)+PrEc\frac{{\mu\:}_{nf}}{{\mu\:}_{f}}\frac{{k}_{f}}{{k}_{nf}}\:{F}^{{˶}^{2}}=0$$

The subsequent conditions are associated to the problem defined in Eqs. (6)–(7).

8 $$\:{F\left(0\right)=0,F}^{{\prime\:}}\left(0\right)=1,\:{F}^{{\prime\:}}\left(\infty\:\right)=0,\:\beta\:\left(0\right)=1,\:\beta\:\left(\infty\:\right)=0$$

The quantities embedded in the model are $\:\lambda\:=G{r}_{x}R{e}_{x}^{-2}$, $\:M=\frac{{\sigma\:}_{f}{B}_{0}^{2}}{a{\rho\:}_{f}},\:Rd=\frac{16{\sigma\:}^{*}{T}_{\infty\:}^{3}}{{k}^{*}{k}_{f}}$, and $\:Pr=\frac{{\nu\:}_{f}}{{\alpha\:}_{f}}$. Further, the SF and Nu are defined by the following ways.

9 $$\:{C}_{f}R{e}_{x}^{\raisebox{1ex}{$1$}\!\left/\:\!\raisebox{-1ex}{$2$}\right.}=\frac{{\mu\:}_{f}}{{\mu\:}_{nf}}{F}^{˶}\left(0\right),\:N{u}_{x}R{e}_{x}^{-\raisebox{1ex}{$1$}\!\left/\:\!\raisebox{-1ex}{$2$}\right.}=-\left(\frac{{k}_{nf}}{{k}_{f}}+Rd\right){\beta\:}^{ˊ}\left(0\right)$$

### Mathematical analysis

The current problem is analyzed using AI based scheme termed as Levenberg Marquardt Back Propagation algorithm. At initial stage, the bvp4c [26], algorithm is implemented and obtained the solutions and created dataset for the NN processing. Parallel to bvp4c (see Refs. [27, 28]), the LMBPS is coded in MATLAB and executed the results. The obtained results are accurate up to $\:1{\boldsymbol{e}}^{-5}$. Further, authenticity is checked through fitting functions, histogram and regressions plot for training, testing, target and then over performance of the scheme. It is clear that the $\:\boldsymbol{R}=1.0$ in all the cases which provides best results.

For the data computation, the RK [29] scheme implemented which works after the reducing the model into appropriate form. For this, the following are the essential steps.

10 $$\:{\boldsymbol{\alpha\:}}_{1}=\boldsymbol{F},\:{\boldsymbol{\alpha\:}}_{2}={\boldsymbol{F}}^{\boldsymbol{{\prime\:}}},\:{\boldsymbol{\alpha\:}}_{3}={\boldsymbol{F}}^{\boldsymbol{{\prime\:}}\boldsymbol{{\prime\:}}},\:{\boldsymbol{\alpha\:}\boldsymbol{{\prime\:}}}_{3}={\boldsymbol{F}}^{\boldsymbol{{\prime\:}}\boldsymbol{{\prime\:}}\boldsymbol{{\prime\:}}},$$

11 $$\:{\boldsymbol{\alpha\:}}_{4}=\boldsymbol{\beta\:},\:{\boldsymbol{\alpha\:}}_{5}={\boldsymbol{\beta\:}}^{\boldsymbol{{\prime\:}}},\:{\boldsymbol{\alpha\:}\boldsymbol{{\prime\:}}}_{5}={\boldsymbol{\beta\:}}^{\boldsymbol{{\prime\:}}\boldsymbol{{\prime\:}}},$$

Now, write the model in the following revised version and then incorporate the expressions from Eqs. (10–11).

12 $$F^{\prime\prime\prime} = \frac{{\mu _{f} }}{{\mu _{{nf}} }}\left[ {\frac{{\rho _{{nf}} }}{{\rho _{f} }}\left\{ {F^{{{\prime }2}} - FF^{\prime\prime}} \right\} - \frac{{\sigma _{{nf}} }}{{\sigma _{f} }}\frac{{\mu _{f} }}{{\mu _{{nf}} }}MF^{\prime} + \frac{{\left( {\rho \alpha } \right)_{{nf}} }}{{\left( {\rho \alpha } \right)_{f} }}\frac{{\mu _{f} }}{{\mu _{{nf}} }}\lambda \beta } \right], $$

13 $$ \beta ^{{{\prime \prime }}} = - \left( {1 + \frac{4}{3}Rd} \right)^{{ - 1}} \left[ {\frac{{\left( {\rho C_{p} } \right)_{{nf}} }}{{\left( {\rho C_{p} } \right)_{f} }}\frac{{k_{f} }}{{k_{{nf}} }}\left( {\Pr F\beta ^{\prime } } \right) + \Pr Ec\frac{{\mu _{{nf}} }}{{\mu _{f} }}\frac{{k_{f} }}{{k_{{nf}} }}F^{{{\prime \prime }2}} } \right], $$

14 $$\:{\boldsymbol{\alpha\:}\boldsymbol{{\prime\:}}}_{3}=\frac{{\mu\:}_{f}}{{\mu\:}_{nf}}\left[\frac{{\rho\:}_{nf}}{{\rho\:}_{f}}\left\{{{\boldsymbol{\alpha\:}}_{2}}^{2}-{\boldsymbol{\alpha\:}}_{1}{\boldsymbol{\alpha\:}}_{3}\right\}-\frac{{\sigma\:}_{nf}}{{\sigma\:}_{f}}\:\frac{{\mu\:}_{f}}{{\mu\:}_{nf}}M{\boldsymbol{\alpha\:}}_{2}+\frac{{\left(\rho\:\alpha\:\right)}_{nf}}{{\left(\rho\:\alpha\:\right)}_{f}}\:\frac{{\mu\:}_{f}}{{\mu\:}_{nf}}\lambda\:{\boldsymbol{\alpha\:}}_{4}\right],$$

15 $$\:{\boldsymbol{\alpha\:}\boldsymbol{{\prime\:}}}_{5}=-{\left(1+\frac{4}{3}Rd\right)}^{-1}\left[\frac{{\left(\rho\:{C}_{p}\right)}_{nf}}{{\left(\rho\:{C}_{p}\right)}_{f}}\:\frac{{k}_{f}}{{k}_{nf}}\left(Pr{\boldsymbol{\alpha\:}}_{1}{\boldsymbol{\alpha\:}}_{5}\right)+PrEc\frac{{\mu\:}_{nf}}{{\mu\:}_{f}}\frac{{k}_{f}}{{k}_{nf}}\:{{\boldsymbol{\alpha\:}}_{3}}^{2}\right],$$

The problem in Eqs. (14)–(15) is then treated via bvp4c with good accuracy.

**Fig. 2:**
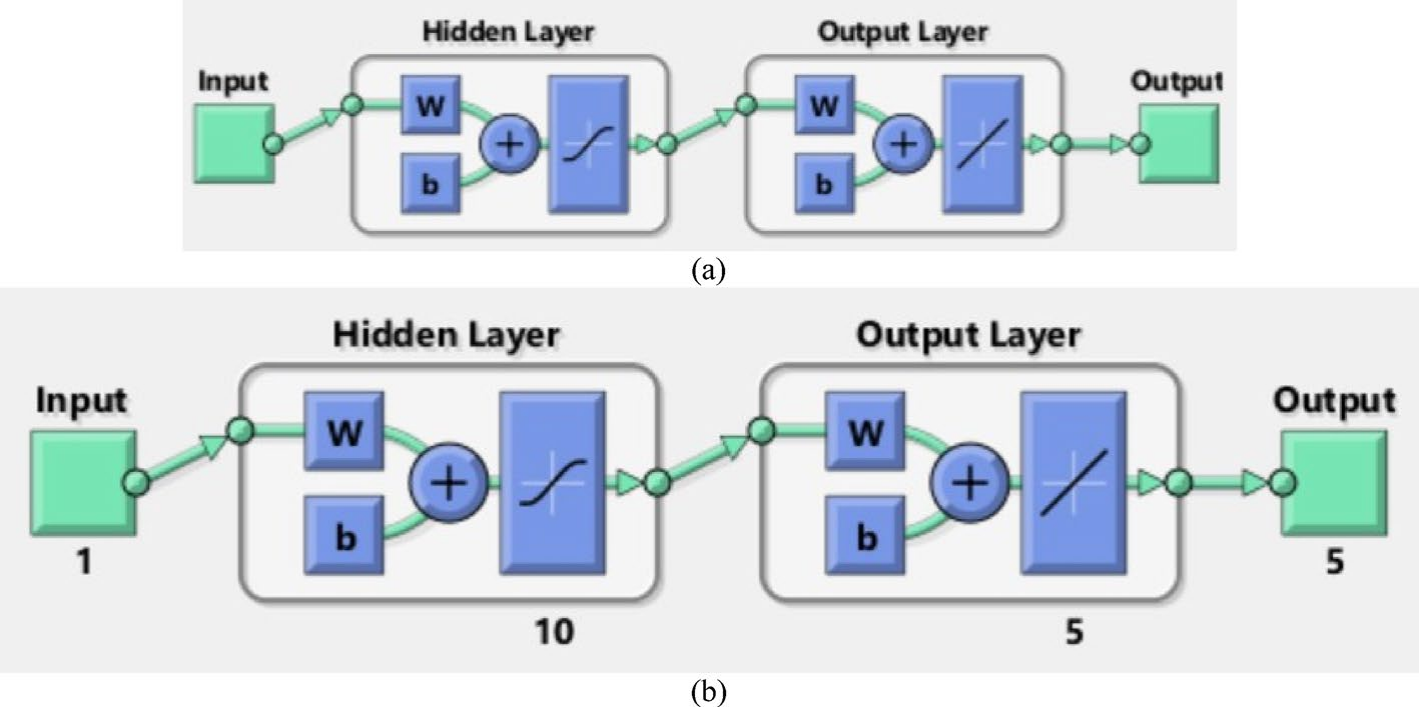
The ANN network for LMBPS

The NN tool is designed in Fig. 2a which shows the two player LMBPS which leads to single output. Moreover, the Fig. 2b presenting the architecture performed for the present results. For good accuracy, the hidden layer comprises 10 neurons and the output layer has 5 neurons. Thus, the results in the next section are subject to mentioned number of neural values.

## Results interpretation

This section comprises a detailed discussion about the problem outcomes due to change in the physical ranges. This provides the thermal and velocity behaviour of nanofluid through the surface.

**Fig. 3:**
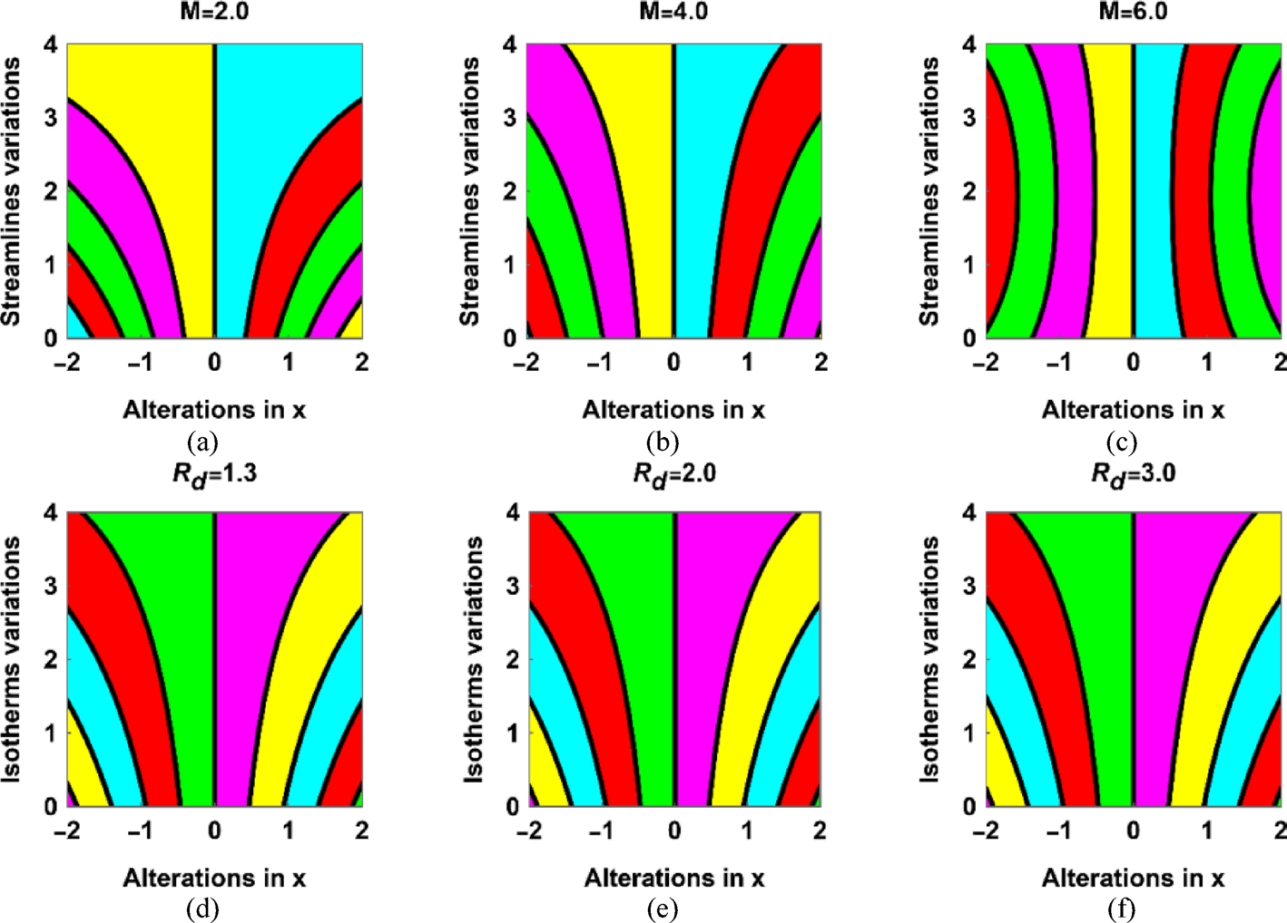
The stream and isotherms contour patterns at different stages

The stream’s contours and isotherms change their behaviour due to fluctuations in the parameters. Figure 3 reveals that the stream contours bend towards the horizontal axis as the strength of $\:M$ depreciated. The Hartmann number increases from 2.0 to 6.0 in Fig. 3a–c which shows the larger magnetic strength, the stream contours start expanding towards $\:x=0.$ In Fig. 3d–f, the heat lines are demonstrated for $\:R=1.3,\:\mathrm{2.0,3.0}$ with default selection of the other parameters. The heatlines strengthen with increase in the radiation source effects. As, this offers more energy to the system which show the higher intensity of the heatlines.

**Fig. 4:**
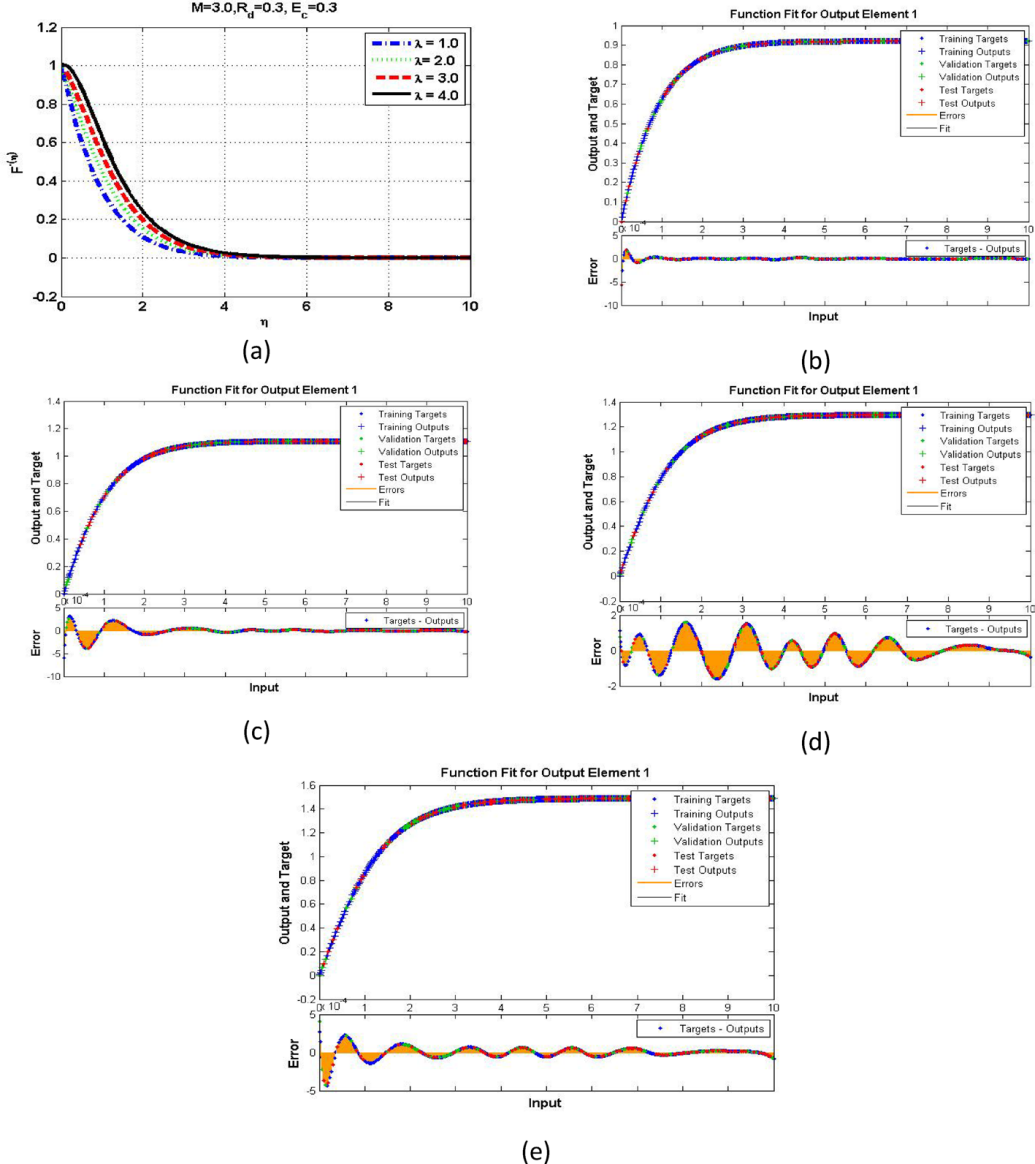
The velocity and function fitting for output element and error for scene 1 to scene 4

**Fig. 5:**
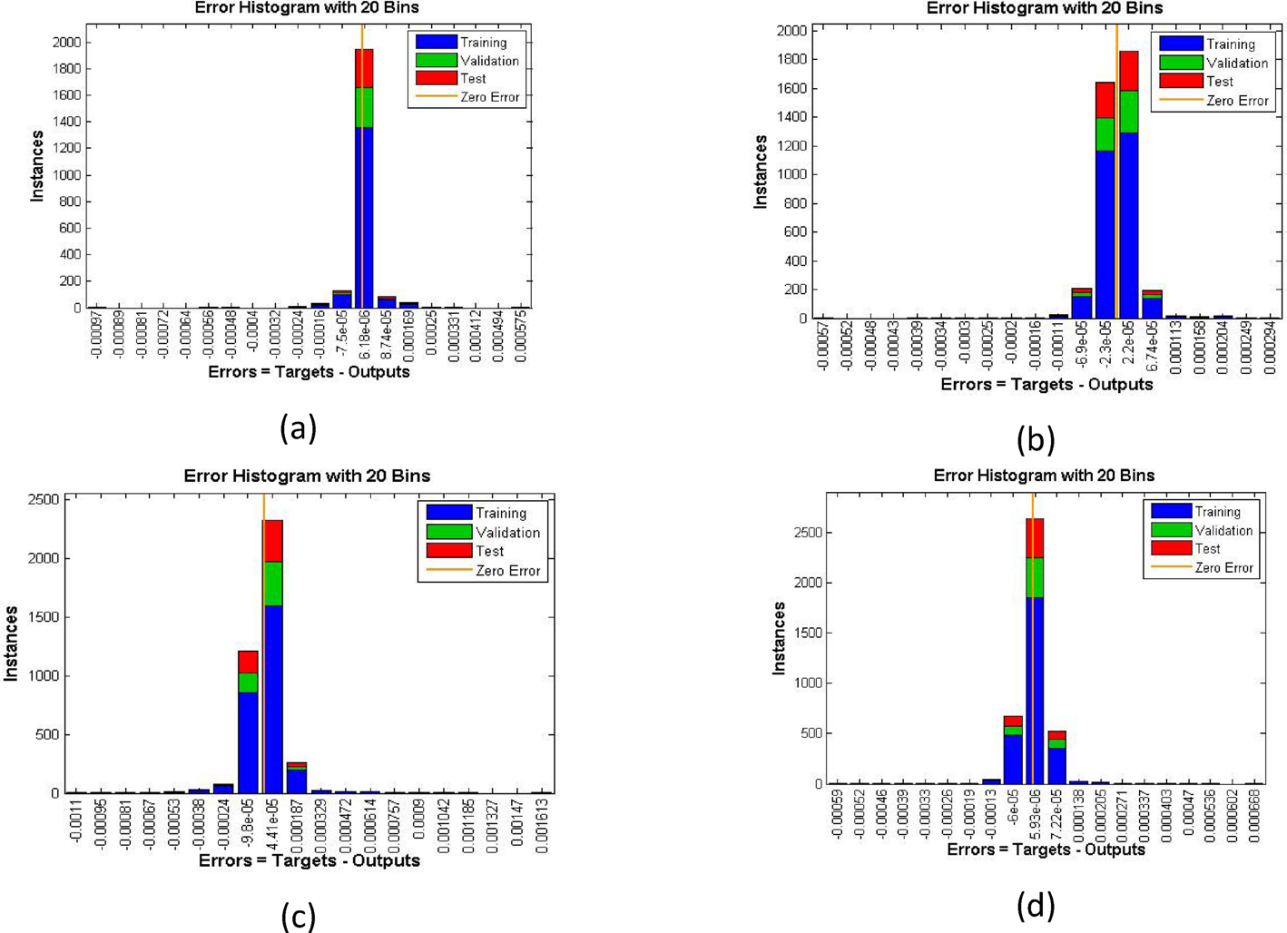
The histogram error estimation for cases 1–4 for Fig. 4

**Fig. 6:**
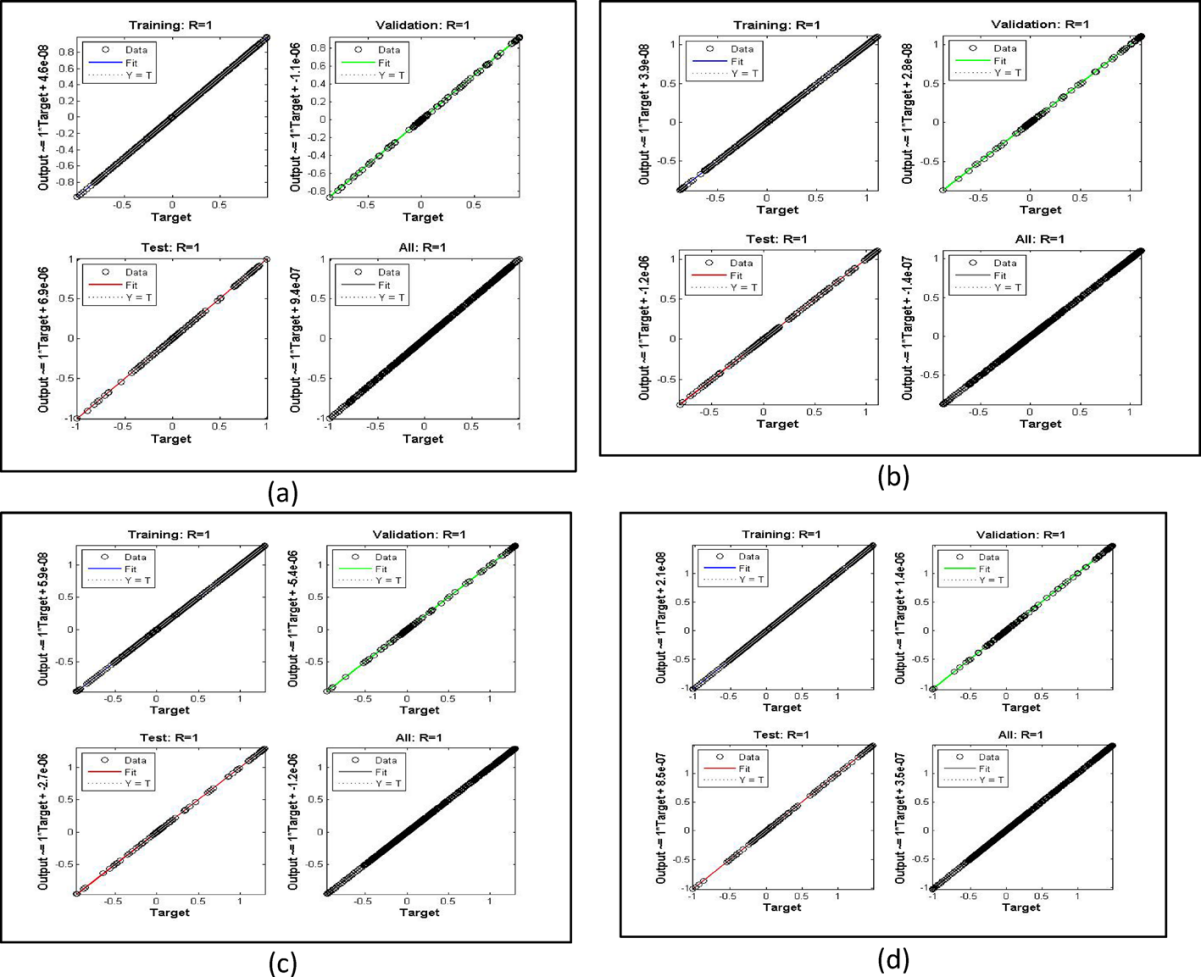
The regressions error estimation for cases 1–4 for Fig. 4

The velocity response due to Grashof number effects is organized in Fig. 4a. The outcomes obtained for multiple cases corresponding to the values $\:\lambda\:=\mathrm{1.0,2.0,3.0,4.0}$. In rest of the parts, the fitting functions and error are also plotted to authenticate the results and the scheme accuracy. The velocity drops due to increasing Grashof effects. Physically, this parameters signify the gravity influence on the fluid movement which attracts the particles downward. Thus, the movement gets slow and approaches to 1 which describes that the BC fulfills at the surface. The ambient portion of the flow is observed beyond $\:\eta\:=4.0$ and after that the fluid approaches to its stable state as $\:{F}^{{\prime\:}}\left(\infty\:\right)=0$. The fitting functions in Fig. 4b–e showing good agreement with multiple testing and training states. Further, Figs. 5 and 6 provided that the histogram error is well and the regressions in training and testing and fit are aligned and $\:R=1.0$ which shows best performance of the scheme.

**Fig. 7:**
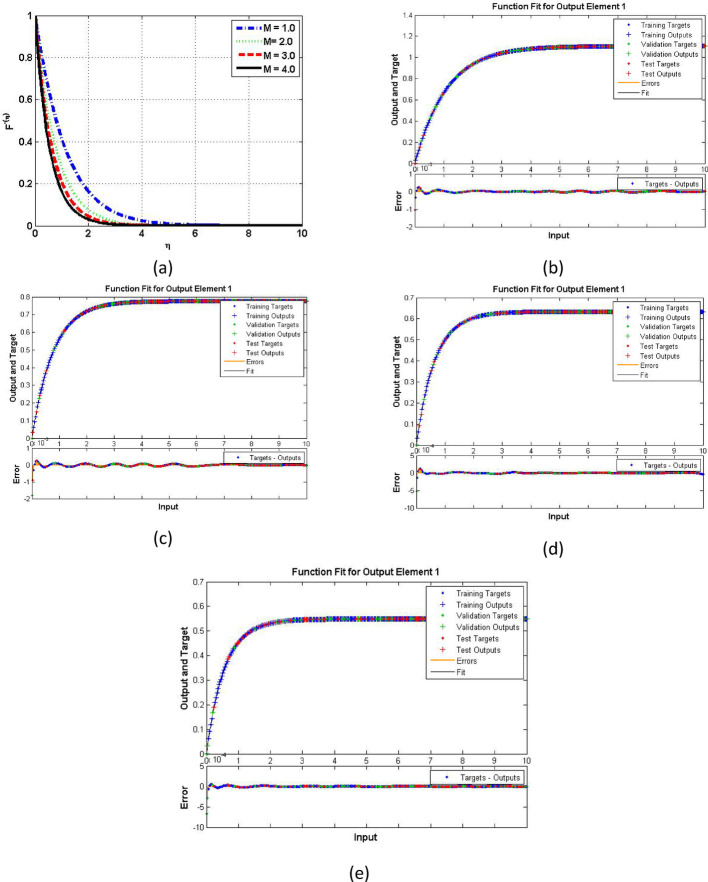
Magnetic field influence in the velocity behaviour

**Fig. 8:**
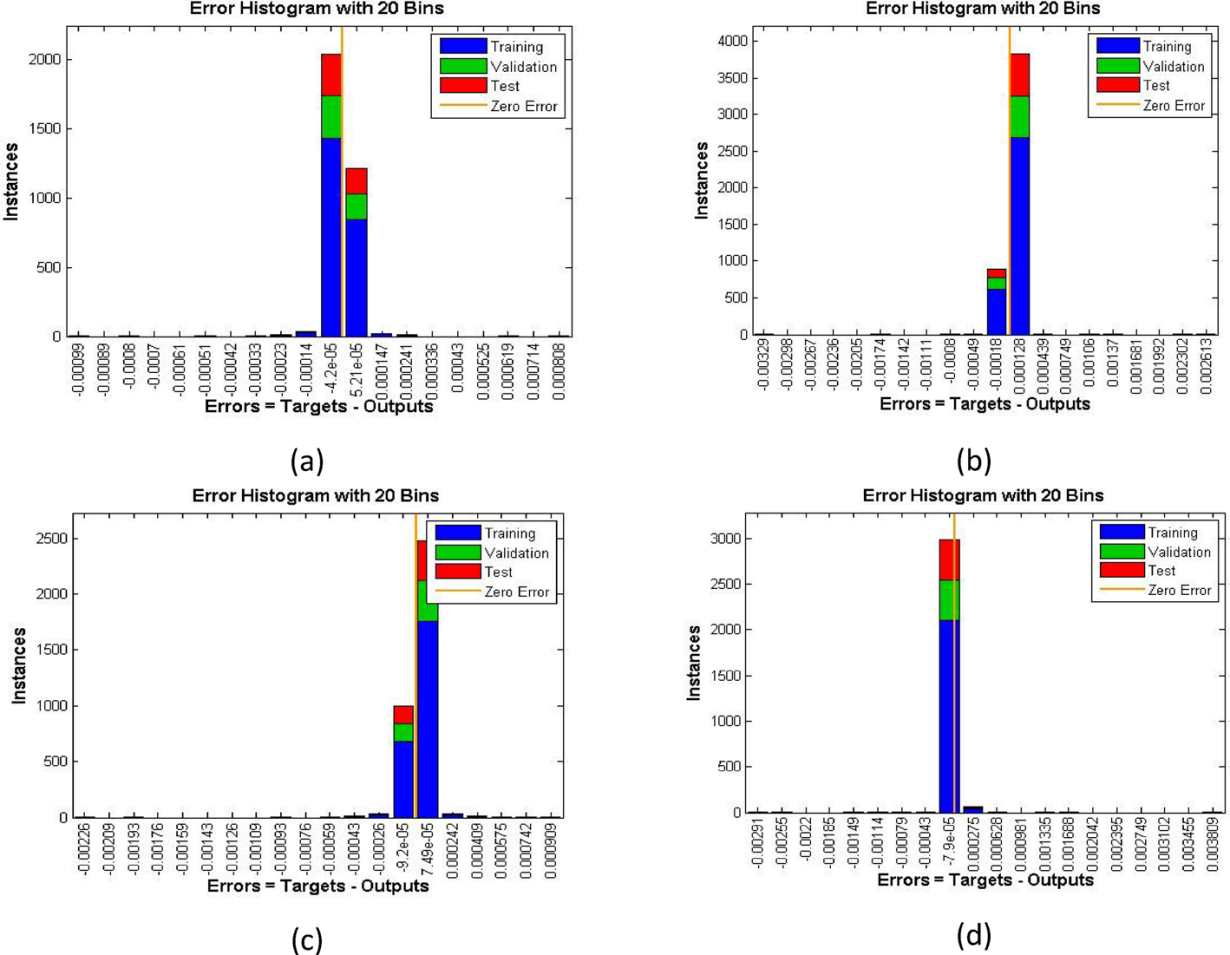
Histogram estimation for increasing $\:M$ for velocity case

**Fig. 9:**
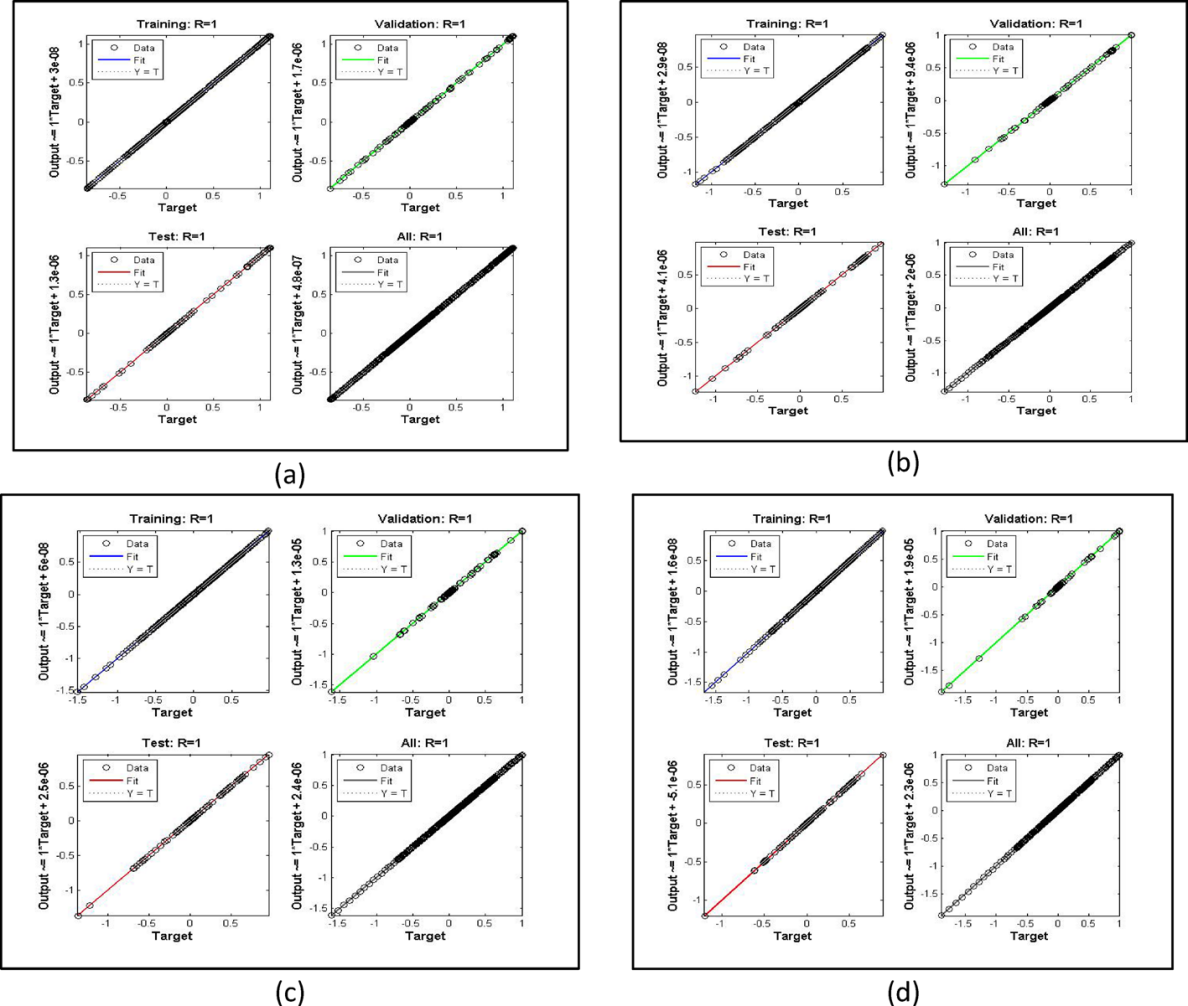
Regressions estimation for increasing $\:M$ for velocity case

**Fig. 10:**
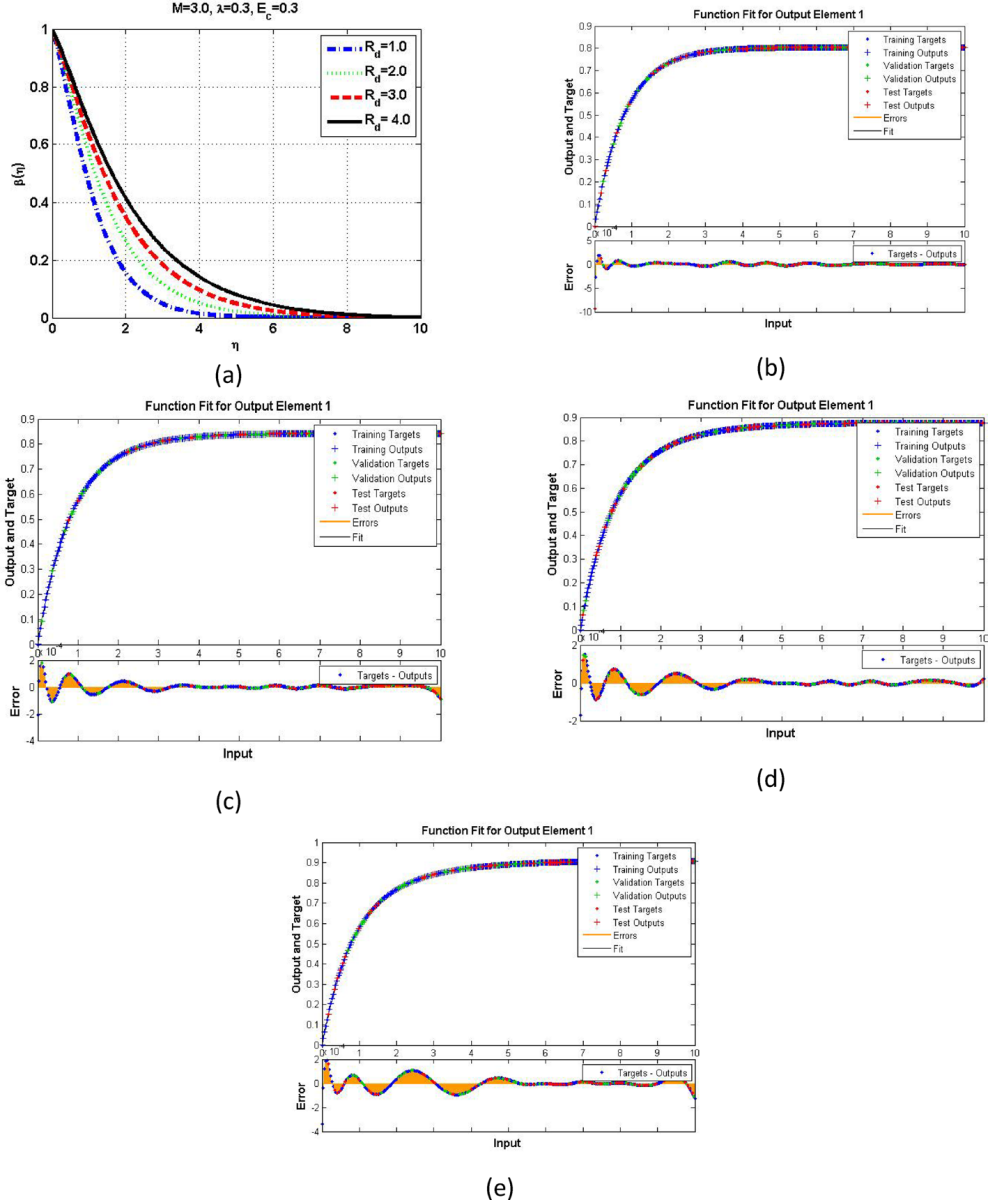
Functions fitting increasing $\:Rd$ for temperature case

**Fig. 11:**
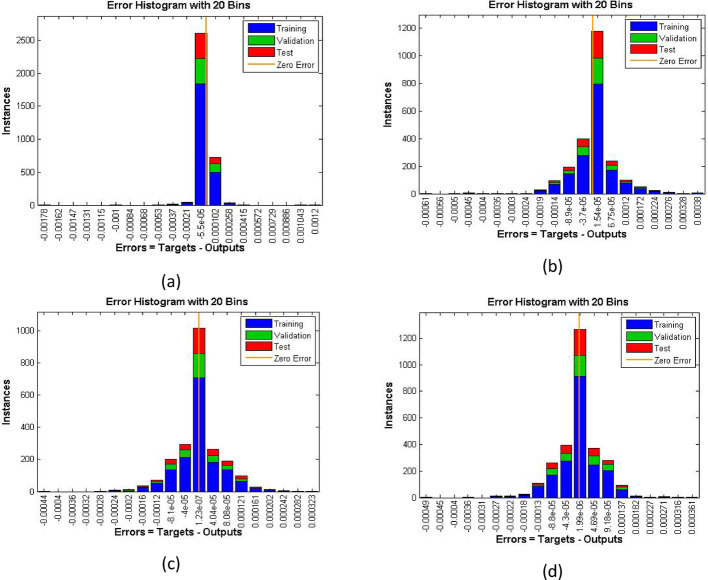
Functions fitting increasing $\:Rd$ for temperature case

**Fig. 12:**
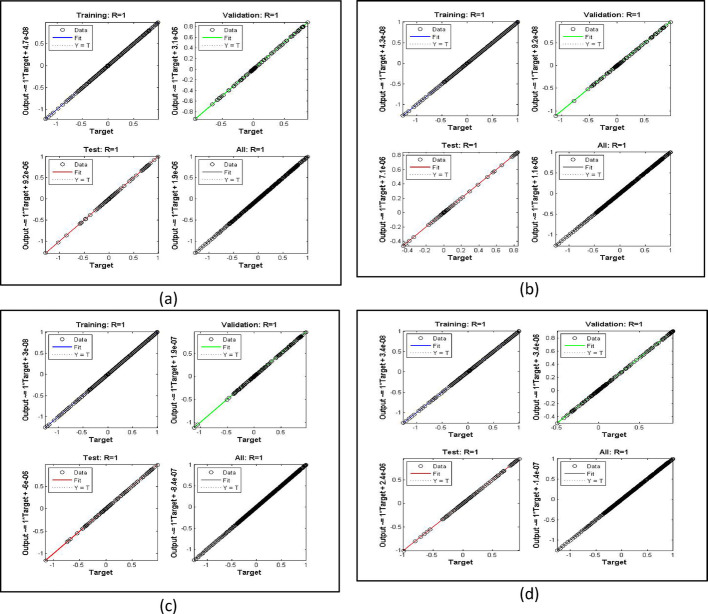
Regression analysis for increasing $\:Rd$ for temperature case

Figures 7, 8 and 9 demonstrating the temperature efficiency for $\:M$ and related fitting functions, histogram and regressions, respectively. Higher the $\:M$ values provide the low temperature of nanofluid. As, the magnetic field gets strengthen it produces Lorentz forces in the flow field which opposes the movement of nanofluid through the flowing region. Applying robust magnetic field helps to control the movement which is good from practical point of view. Further, the TBL depreciated as the $\:M$ varies from 1.0 to 4.0. The fitting functions (Fig. 7), histogram error estimation (Fig. 8), and regression fitting (Fig. 9) demonstrating excellent accuracy of the employed scheme. The error is considerable which gives the reliability of the obtained results. Figures 10, 11 and 12 highlighting the impacts of thermal radiations ($\:Rd$) on the trends of $\:\beta\:\left(\eta\:\right)$. The result shows that radiation source is helpful to acquire good thermal transport and is natural source. Physically, higher strength of radiations boosts the internal energy of the fluid which directly enhances the performance. Further, the excellent errors are observed for this case also.

**Fig. 13:**
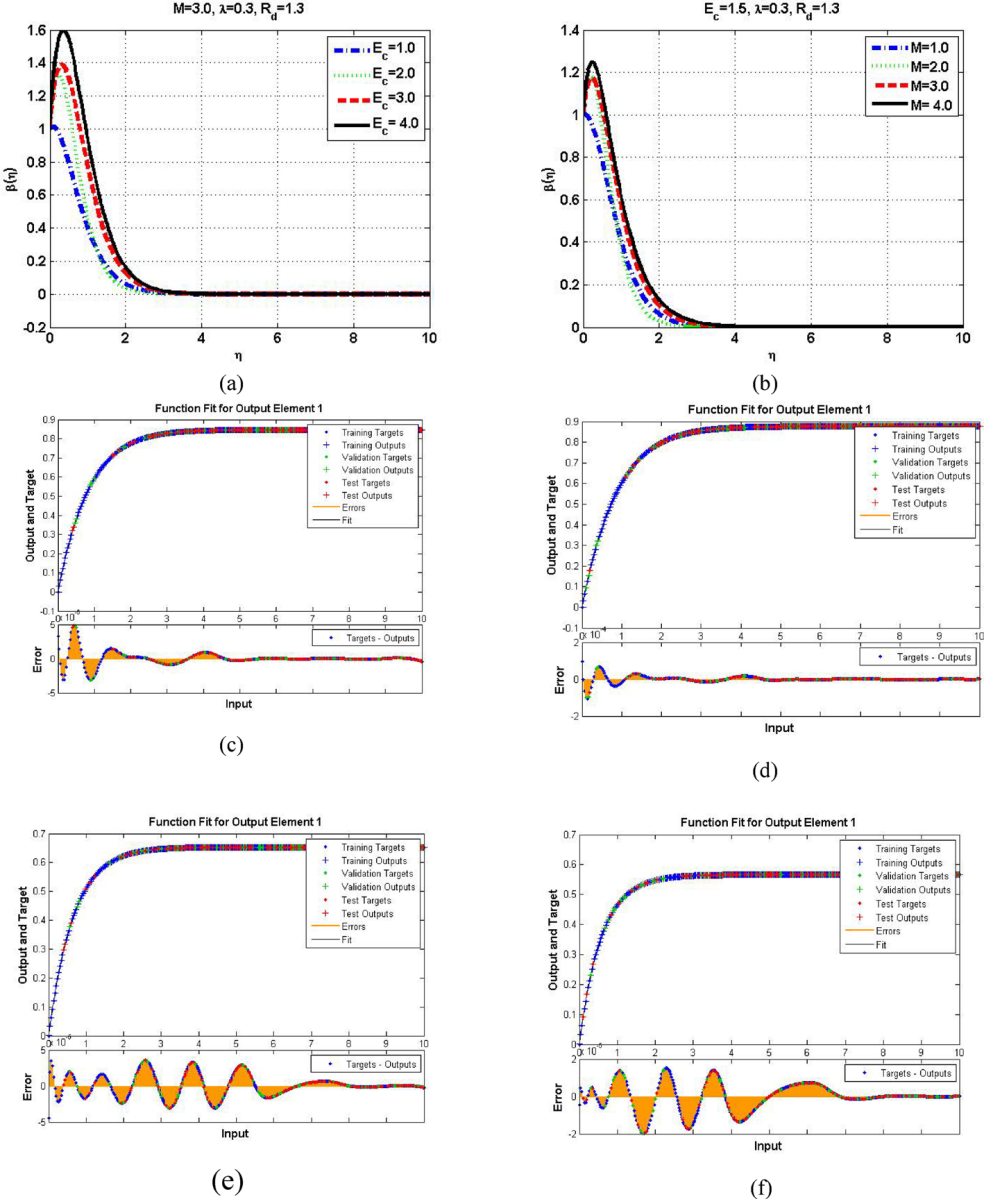
Temperature performance for $\:Ec$ and $\:M$

Figure 13 provides the temperature trends for Eckert and $\:M$ numbers along with error scenarios for multiple cases. The TBL drops for both the physical scenarios while small region is noticed for increasing dissipation effects. Physically, increase in Eckert number values make disturbance among the molecules which enhances the internal kinetic energy. As a results, the temperature of the fluid goes higher. The optimized trends are observed at the surface which gives the signal of strong dissipation influence there. In contrast, the Lorentz forces decline the motion which control the temperature. Further, the error is observed very good as furnished in Fig. 13c–f, respectively.

Table 1 describes variation of skin friction against different physical parameters according to updated model. Increase in NPs concentration $\:\phi\:$ and magnetic field effects $\:M$ enhances the skin friction on the surface. Physically, increasing nanoparticles ratio in base liquid enhances density due to which mass per unit volume in the fluid rises. The fluid flow resistance upsurges and the movement become slow which allows more drag on the surface. On the other hand, strengthen Hartmann number directly affects the fluid motion over the surface and resists it due to Lorentz forces. Thus, these parameters provided drag enhancement due to their physical impacts on the flow regime. Also, the mixed convection and Eckert numbers help to minimize the drag force on the functional surface.

Table 2 illustrates the variations of Nusselt number against physical parameters. Significant increase in the heat transfer rate is achieved for $\:\phi\:,\:\lambda\:$ and $\:Rd$. Physically, these parameters promoting internal energy of the fluid medium by strengthening thermal conductivity due to extra NPs concentration, mixed convection and addition of energy from surrounding to the fluid system. In the view of these physical interactions, the heat transfer enhances in the nanofluid. The $\:M$ and $\:Ec$ examined to be useful to regulate the fluid at low heat transfer rate under considered physical situation.

**Table 1:** Variation of shear drag against physical parameters

Parameters					SF
	*M*		*Rd*	*Ec*	$F^{{{\prime \prime }}} \left( 0 \right)$
0.02					− 2.1064
0.04					− 1.7423
0.06					− 1.6115
0.08					− 1.5416
0.02	0.1				− 1.3974
	0.2				− 1.3477
	0.3				− 1.2968
	0.4				− 1.2446
	0.1	0.1			− 0.1845
		0.2			− 0.2690
		0.3			− 0.3544
		0.4			− 0.4407
		0.1	0.1		− 0.1845
			0.2		− 0.1838
			0.3		− 0.1832
			0.4		− 0.1826
			0.1	0.1	− 0.1845
				0.2	− 0.1852
				0.3	− 0.1859
				0.4	− 0.1866

**Table 2:** Variation of Nu against physical parameters

Parameters					Nu
	*M*		*Rd*	*Ec*	
0.001					6.0386
0.002					8.0855
0.003					11.2095
0.004					15.8051
0.001	0.1				0.4088
	0.2				0.4057
	0.3				0.4024
	0.4				0.3989
	0.1	0.1			0.4035
		0.2			0.4088
		0.3			0.4137
		0.4			0.4182
		0.1	0.1		0.4035
			0.2		0.4545
			0.3		0.4964
			0.4		0.5315
			0.1	0.1	0.4035
				0.2	0.3154
				0.3	0.2271
				0.4	0.1386

In order to authenticate the study, a comparison is provided in Table 3 with the results of Abdelgaber et al. [30]. The computation carried when $\:{\eta\:}_{\infty\:}\to\:10$ and the current findings become well aligned with literature data which gives reliability of the study. These computation for $- F^{{{\prime \prime }}} \left( 0 \right)$ performed when mixed convection and concentration factors approach to zero to meet the better outcomes of the model.

**Table 3:** Comparison of the current results for$- F^{{{\prime \prime }}} \left( 0 \right)$with previous literature under$\:\lambda\:=\phi\:=0$

$\:M$	Abdelgaber et al. [30]	Present
0.0	0.999993	0.999899
0.5	1.224690	1.224589
1.0	1.414020	1.414016
1.5	1.580640	1.580631

## Conclusions

The study of nanofluid through a surface associated to significant physical effects is conducted using LMBPS and examined that:

- The movement of the fluid controlled by increasing $\:\lambda\:=\mathrm{1.0,2.0,3.0,4.0}$ and vanishes towards the freestream after $\:\eta\:=4.0$. Thus, increasing mixed convection helps to control the movement which beneficial from engineering point of view where decreased fluid motion is essential.
- Increasing the Lorentz forces strength ($\:M=\mathrm{1.0,2.0,3.0,4.0}$), the movement can be controlled which is a good physical tool for purification industries.
- The thermal radiations ($\:Rd=\mathrm{1.0,2.0,3.0,4.0}$) provided external energy to the system which plays the role of catalysis and improves the temperature significantly.
- Addition of Eckert number and magnetic field also observed good for thermal enhancement but, the $\:Ec$ playing major role in this regard.
- The $\:Nu$ (heat transfer rate) shown improvement for radiations and dissipation energy influence and transfer more heat on the functional surface.
- The remarkable accuracy of the ANNS is achieved through fitting functions, error histogram and regression analysis.

The control in the fluid movement is excellent for products purifications and increase in the thermal behaviour would be good for heat transport applications. The predictive ranges would help to maintain the temperature and fluid movement for practical implications. Further, the insights of mixed convection [31] for hybrid nanofluid’s shape factors [32], Corcione effects [33], slip effects for thermal and momentum [34], blood based nanofluid [35], heat source, modified BCs and CCHFM are the new aspects of the study. In future, the current work can be extended using these physical aspects. Moreover, extending the work towards non-Newtonian fluid particularly for Cross and Carreau models would be of great interest for engineering heat and mass transfer applications.

## Data Availability

The manuscript has no associated data.

## Abbreviations

$v,u$: Components of velocity ($m/s$)
$$T$$: Temperature ($$K$$)
$$g$$: Gravitational acceleration ($$m/{s}^{2}$$)
$${\sigma}_{nf}$$: Electrical conductivity ($$S/m$$)
$${\mu}_{nf}$$: Dynamic viscosity ($$Ns/{m}^{2}$$)
$${k}_{nf}$$: Thermal conductivity ($$W/mK$$)
$$M$$: Hartmann number
$\lambda$: Mixed convection number
$$Rd$$: Radiation number
$$Pr$$: Prandtl number
$$Ec$$: Eckert number
$$\eta $$: Dimensionless variable
$$F{\prime}$$: Dimensionless velocity
$$\beta $$: Dimensionless temperature
